# Decreased Insulin-Stimulated ATP Synthesis and Phosphate Transport in Muscle of Insulin-Resistant Offspring of Type 2 Diabetic Parents

**DOI:** 10.1371/journal.pmed.0020233

**Published:** 2005-08-16

**Authors:** Kitt F Petersen, Sylvie Dufour, Gerald I Shulman

**Affiliations:** 1Department of Internal Medicine, Yale University School of Medicine, New Haven, Connecticut, United States of America,; **2**Department of Cellular and Molecular Physiology, Yale University School of Medicine, New Haven, Connecticut, United States of America,; **3** Howard Hughes Medical Institute, Yale University School of Medicine, New Haven, Connecticut, United States of America; University of Warwick Medical SchoolUnited Kingdom

## Abstract

**Background:**

Insulin resistance is the best predictor for the development of type 2 diabetes. Recent studies have shown that young, lean, insulin-resistant (IR) offspring of parents with type 2 diabetes have reduced basal rates of muscle mitochondrial phosphorylation activity associated with increased intramyocellular lipid (IMCL) content, which in turn blocks insulin signaling and insulin action in muscle. In order to further characterize mitochondrial activity in these individuals, we examined insulin-stimulated rates of adenosine triphosphate (ATP) synthesis and phosphate transport in skeletal muscle in a similar cohort of participants.

**Methods and Findings:**

Rates of insulin-stimulated muscle mitochondrial ATP synthase flux and insulin-stimulated increases in concentrations of intramyocellular inorganic phosphate (P_i_) were assessed by ^31^P magnetic resonance spectroscopy (MRS) in healthy, lean, IR offspring of parents with type 2 diabetes and healthy, lean control participants with normal insulin sensitivity. IMCL content in the soleus muscle of all participants was assessed by ^1^H MRS. During a hyperinsulinemic-euglycemic clamp, rates of insulin-stimulated glucose uptake were decreased by approximately 50% in the IR offspring compared to the control participants (*p* = 0.007 versus controls) and were associated with an approximately 2-fold increase in IMCL content (*p* < 0.006 versus controls). In the control participants rates of ATP synthesis increased by approximately 90% during the hyperinsulinemic-euglycemic clamp. In contrast, insulin-stimulated rates of muscle mitochondrial ATP synthesis increased by only 5% in the IR offspring (*p* = 0.001 versus controls) and was associated with a severe reduction of insulin-stimulated increases in the intramyocellular P_i_ concentrations (IR offspring: 4.7% ± 1.9% versus controls: 19.3% ± 5.7%; *p* = 0.03). Insulin-induced increases in intramyocellular P_i_ concentrations correlated well with insulin-stimulated increases in rates of ATP synthesis (*r* = 0.67; *p* = 0.008).

**Conclusions:**

These data demonstrate that insulin-stimulated rates of mitochondrial ATP synthesis are reduced in IR offspring of parents with type 2 diabetes. Furthermore, these IR offspring also have impaired insulin-stimulated phosphate transport in muscle, which may contribute to their defects in insulin-stimulated rates of mitochondrial ATP synthesis.

## Introduction

Type 2 diabetes affects about 171 million people worldwide, and the number of people likely to be affected by diabetes is expected to double by 2030 [1,2]. Diabetes develops when resistance to insulin action combines with impaired insulin secretion, resulting in hyperglycemia. Cross-sectional studies have demonstrated the presence of insulin resistance in virtually all patients with type 2 diabetes, and prospective studies have demonstrated the presence of insulin resistance one to two decades prior to the onset of the disease [3,4]. In addition, insulin resistance in the insulin-resistant (IR) offspring of parents with type 2 diabetes has been shown to be the best predictor for the later development of the disease [5]. Although the mechanism for their insulin resistance is unknown, previous studies have demonstrated a strong relationship between dysregulated intracellular fatty acid metabolism and insulin resistance in skeletal muscle [6–8]. Increases in certain intramyocellular lipid (IMCL) metabolites such as acyl CoAs and diacylglycerol have in turn been shown to block insulin signaling by activating a serine kinase cascade [9–12], resulting in reduced insulin-stimulated muscle glucose transport activity [10] and decreased muscle glycogen synthesis [13,14]. Recent combined ^1^H and ^31^P magnetic resonance spectroscopy (MRS) studies have demonstrated an approximately 2-fold increase in the IMCL content in a group of healthy, young, lean, IR offspring of parents with type 2 diabetes associated with a 30% reduction in basal rates of muscle mitochondrial adenosine triphosphate (ATP) production. These data suggest that defects in mitochondrial activity, due to reduction in mitochondrial content and/or function, may be an important contributing factor to their increased IMCL content and insulin resistance [15]. Consistent with these functional MRS data two recent gene microarray studies have found a coordinated reduction in peroxisome proliferator-activated receptor γ coactivator 1 responsive genes that are involved in mitochondrial oxidative phosphorylation activity in muscle biopsy samples obtained from obese participants with type 2 diabetes [16,17] and their overweight first-degree relatives [17].

In order to further characterize mitochondrial function in these individuals we measured insulin-stimulated rates of muscle mitochondrial ATP synthesis and changes in intramyocellular inorganic phosphate (P_i_) concentrations using ^31^P MRS in a similar group of young, lean, IR offspring of parents with type 2 diabetes and body mass index (BMI)–age–activity-matched control participants. These individuals are ideal for examining the earliest defects responsible for the pathogenesis of insulin resistance since, in contrast to patients with diabetes, they are young, lean, healthy, and they have none of the other confounding factors that are likely to be present in patients with type 2 diabetes.

## Methods

### Participants

All volunteers were prescreened to be healthy, taking no medications, lean, nonsmoking, of normal birth weight (>2.25 kg), and sedentary, as defined by an activity index questionnaire [18]. IR offspring (four male and three female) had at least one parent or grandparent with type 2 diabetes and at least one other family member with type 2 diabetes. All qualifying participants underwent a complete medical history and physical examination along with blood tests to verify normal: blood and platelet count, electrolytes, aspartate amino transferase, alanine amino transferase, blood urea nitrogen, creatinine, cholesterol, and triglyceride. IR offspring were defined by an insulin (240 pmol/m^2^/min)–stimulated glucose infusion rate <4.85 mg/kg/min, whereas the control participants were defined by a rate of insulin-stimulated glucose uptake >6 mg/kg/min.

Written consent was obtained from each participant after the purpose, nature, and potential complications of the studies were explained. The protocol was approved by the Yale University Human Investigation Committee.

### Diet and Study Preparation

Participants were instructed to abstain from any exercise and to eat a regular, weight maintenance diet containing at least 150 g of carbohydrate per day for the 3 d prior to the studies. To minimize changes in ovarian hormonal effects on glucose metabolism, the female participants were studied during the follicular phase (days 0–12) of the menstrual cycle [19].

### Hyperinsulinemic-Euglycemic Clamp

Participants were admitted to the Yale University–New Haven Hospital General Clinical Research Center the evening before the studies and remained fasting from 10 p.m. until the end of the study the next day. At 6 a.m. antecubital IV catheters were inserted, and at 7 a.m. the participants were placed inside the 2.1T magnetic resonance spectrometer (Biospec Spectrometer, Bruker Instruments, Billerica, Massachusetts, United States) with the right calf positioned over the ^31^P receiver coil. After baseline ^31^P MRS measurements of ATP synthesis flux and muscle intramyocellular P_i_ concentrations (described below), a primed-continuous insulin infusion was begun (240 pmol/m^2^/min) to raise plasma insulin concentrations to approximately 480 pmol/l and to maintain them at this level throughout the remaining 150 min of the clamp. Blood was collected every 5 min for determination of plasma glucose concentrations, and a variable intravenous infusion of dextrose (200 g/l) was administered to raise plasma glucose concentrations to 100 mg/dl and maintained at this concentration until the end of the clamp.

### Metabolites and Hormones

Plasma glucose concentrations were measured using a Glucose Analyzer II (Beckman Instruments, Fullerton, California, United States). Plasma concentrations of insulin were measured using double-antibody radioimmunoassay kits (Linco, St. Louis, Missouri, United States). Plasma fatty acid concentrations were determined using a microfluorimetric method [20]. Urine nitrogen content was measured at the Mayo Medical Laboratories (Rochester, Minnesota, United States).

### ^31^P MRS

Rates of mitochondrial phosphorylation activity were assessed by ^31^P MRS saturation transfer performed at 36.31 MHz using a flat, concentric probe made of a 9-cm-diameter inner coil (for ^31^P) and a 13-cm outer coil tuned to proton frequency for scout imaging and shimming as previously described [15,21]. Unidirectional rates of ATP synthesis were measured at baseline and during the insulin clamp, using the saturation transfer method applied to the exchange between intracellular phosphate and ATP [21]. The steady-state intramyocellular P_i_ magnetization was measured in the presence of a selective irradiation of the γ resonance of ATP and compared to the equilibrium intracellular phosphate magnetization in a control spectrum (without irradiation of γ ATP) [21]. Total acquisition time for ^31^P spectra was about 120 min (from *t* = 30 to 150 min of the clamp).

### ^1^H MRS of IMCL Content

On a separate day, after a 12-h fast, all participants were transported by wheelchair to the Yale Magnetic Resonance Center and localized ^1^H MRS of the soleus muscle to assess IMCL content were acquired on a 2.1T Biospec as previously described [22].

### Indirect Calorimetry

Rates of whole-body energy expenditure and glucose and fat oxidation were assessed by indirect calorimetry (Deltratrack Metabolic Monitor, Sensormedics, Anaheim, California, United States) during the last 20 min of the baseline period and during the last 20 min of the clamp [23].

### Calculations

#### Hyperinsulinemic-euglycemic clamp

The rates of glucose infusion were calculated in 15-min blocks from 60–150 min during the insulin clamp. The data were corrected for urinary glucose loss and glucose space and were expressed as milligrams glucose metabolized per kilogram body weight per minute.

#### Indirect calorimetry

Basal and insulin-stimulated glucose and lipid oxidation rates were measured by the ventilated hood technique, using a Deltatrack Metabolic Monitor (as previously described [24]). The nonprotein respiratory quotients for 100% oxidation of fat and for oxidation of carbohydrates were 0.707 and 1.00, respectively [24]. Nonoxidative glucose metabolism was calculated by subtracting the amount of glucose oxidized from the total amount of glucose infused.

#### Statistical analyses

Statistical analyses were performed using the unpaired *t*-test to detect statistical differences between control and IR offspring, and the paired student *t*-tests were performed to test for interparticipant differences. A *p*-value (two-sided) of <0.05 was considered significant. All data are expressed as mean ± SEM.

## Results

### Participant Characteristics

The control participants and IR offspring were group matched for age, weight, height, BMI, and activity (Table 1). All participants were lean, nonsmoking, and had a sedentary lifestyle without regular participation in sports activities.

**Table 1:**
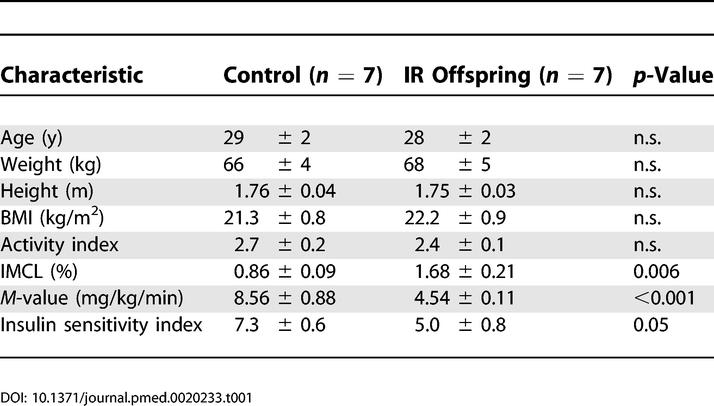
Characteristics of the Two Groups of Participants

### Hyperinsulinemic-Euglycemic Clamp

Rates of insulin-stimulated glucose metabolism were approximately 50% lower in the IR offspring compared to the control participants (control: 8.56 ± 0.88 mg/[kg/min] and IR offspring: 4.54 ± 0.11 mg/[kg/min]; *p* < 0.001) (Figure 1A). This reduction in insulin-stimulated rate of whole-body glucose metabolism was associated with an approximately 90% increase in the IMCL content in the IR offspring (1.68% ± 0.21% versus controls: 0.86 ± 0.09%, *p* = 0.006) and could mostly be attributed to an approximately 60% reduction (*p* < 0.001) in nonoxidative glucose disposal (Table 2).

**Figure 1:**
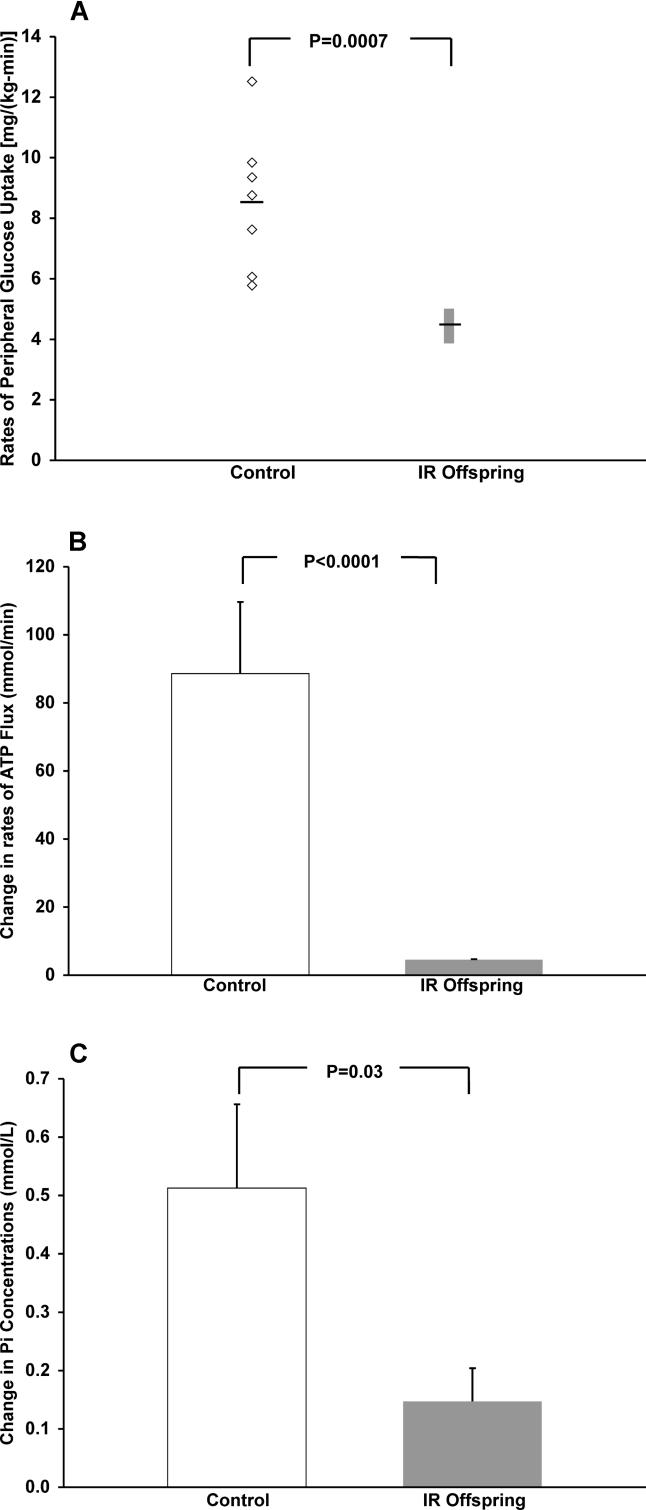
Effect of Insulin Stimulation (A) Rates of whole-body glucose metabolism, (B) rates of muscle mitochondrial ATP synthesis, and (C) intramyocellular P_i_ concentrations.

**Table 2:**
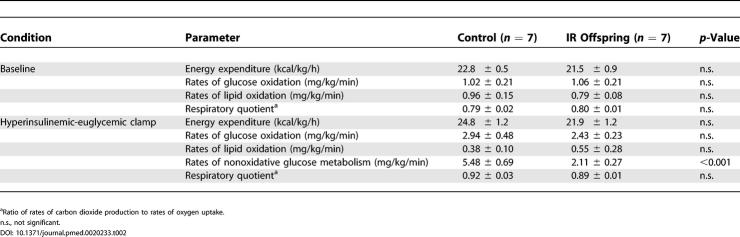
Rates of Energy Expenditure, Glucose and Lipid Oxidation, and Respiratory Quotient Values in the Basal State and during the Hyperinsulinemic-Euglycemic Clamp in Control Individuals and IR Offspring

### ^31^P MRS

During insulin stimulation, rates of mitochondrial phosphorylation activity increased by approximately 90% in the control participants (Figure 1B). In contrast, insulin-stimulated ATP synthase flux was severely blunted in the IR offspring (ΔATP flux: 5% ± 2%; *p* = 0.001) (Figure 1B). During insulin stimulation concentrations of intramyocellular P_i_ increased by 19.3% ± 5.7% in the control participants, whereas insulin-stimulated increases in intramyocellular Pi concentrations were also severely blunted in the IR offspring (ΔPi: 4.7% ± 1.9%; *p* = 0.03 versus controls) (Figure 1C). There was a strong correlation between insulin-induced increases in intramyocellular P_i_ concentrations, and the insulin-stimulated increases in rates of ATP synthesis (*r* = 0.67; *p* = 0.008). In order to determine whether or not these changes in intramyocellular P_i_ might be attributable to insulin-induced alterations in phosphocreatinine (PCr) metabolism, we also assessed the PCr/ATP ratio before and during the clamp in both groups of participants. We found no changes in the PCr/ATP ratio during the hyperinsulinemic-euglycemic clamp compared to the basal period in either the control participants (basal: 4.36 ± 0.06 versus clamp: 4.30 ± 0.31) or IR offspring (basal: 3.82 ± 0.17 versus clamp: 3.91 ± 0.33).

## Discussion

Recent studies have demonstrated an approximately 30% reduction in basal rates of mitochondrial ATP synthesis in healthy, young, lean, IR offspring of parents with type 2 diabetes, which were associated with an approximately 2-fold increase in IMCL content [15]. We postulated that reductions in mitochondrial activity might predispose these individuals to increased IMCL accumulation, which in turn would lead to defects in insulin signaling and insulin action in skeletal muscle. In order to further characterize the regulation of mitochondrial activity in these individuals, we assessed insulin-stimulated rates of ATP synthesis in a similar group of young, lean, IR offspring as well as in a group of age–BMI–activity-matched control group participants using ^31^P MRS. We found that insulin was a potent stimulator of muscle ATP synthesis in that it increased this flux by 90% over basal rates in the control participants. In contrast, insulin-stimulated rates of ATP synthase flux were severely blunted in the IR offspring where a similar increase in plasma insulin concentrations increased this flux by only 5% over basal rates. These data demonstrate that the defects in basal rates of mitochondrial phosphorylation activity, previously found in the IR offspring, are even more pronounced under insulin-stimulated conditions and are consistent with a recent in vitro study that found reduced insulin-stimulated rates of oxidative phosphorylation activity in isolated mitochondria obtained from muscle biopsy samples of participants with type 2 diabetes [25]. Taken together these data suggest that the observed defect in insulin-stimulated rates of ATP synthesis is a very early defect in the pathogenesis of insulin resistance and type 2 diabetes.

Insulin also stimulates intramyocellular P_i_ transport into skeletal muscle, which accounts for much of its hypophosphatemic effects in vivo. Previous in vitro studies by Polgreen et al. [26] have demonstrated that insulin stimulates Na-dependent intramyocellular P_i_ influx in a mouse myoblast cell line in a relatively rapid manner that is independent of protein synthesis. These authors speculated that insulin regulates intramyocellular P_i_ transport into skeletal muscle by recruitment of intramyocellular P_i_ transporters to the cell surface in a fashion similar to insulin regulation of glucose transport activity in skeletal muscle. Using ^31^P MRS to assess intramyocellular P_i_ content before and during the hyperinsulinemic-euglycemic clamp, we found a marked reduction of insulin-stimulated intramyocellular P_i_ transport into skeletal muscle of the IR offspring. The parallel reductions in insulin-stimulated rates of muscle glucose metabolism, which can mostly be attributed to reduced insulin-stimulated glucose transport activity [27], and reduced insulin-stimulated phosphate transport activity suggests that a common insulin-signaling defect may be responsible for both of these abnormalities [28]. We also observed a strong correlation between insulin-stimulated increases in intramyocellular phosphate concentrations and insulin-stimulated increases in rates of ATP synthesis in control participants and IR offspring. Inorganic phosphate is the substrate for the phosphorylation of adenosine diphosphate to ATP during oxidative phosphorylation, and it is also a putative cytosolic signaling molecule in the regulation of this process [29,30]. Recent in vitro studies in isolated heart mitochondria have demonstrated that inorganic phosphate regulates oxidative phosphorylation by influencing both the proton motive force and the rate of ATP production [31]. Taken together these data suggest that insulin regulation of phosphate transport may be an important mechanism by which insulin regulates ATP synthesis in human skeletal muscle. If this is true, then it is possible that the observed reductions in insulin-stimulated rates of muscle ATP synthesis, which were observed in the IR offspring, may be secondary to the increased IMCL content that results in impaired insulin signaling and decreased insulin-stimulated phosphate transport [9–12]. While it is also possible that reduced insulin-stimulated muscle capillary recruitment [32–34] might be responsible for the observed reductions in insulin stimulation of ATP synthesis and intramyocellular P_i_ transport in the IR offspring, recent studies in humans [27,35–37] have shown that the majority of insulin resistance can be accounted for by a decrement in insulin's stimulatory effects on cellular glucose uptake and that stimulation of blood flow and capillary recruitment plays only a minor role in mediating insulin effects on glucose metabolism in human skeletal muscle. Therefore it appears unlikely that reductions in insulin-stimulated muscle capillary recruitment have a major role in explaining the observed reductions in insulin-stimulated ATP synthesis and intramyocellular P_i_ transport in the IR offspring.

In summary, these data provide further evidence of mitochondrial dysfunction in skeletal muscle of IR offspring of parents with type 2 diabetes. These individuals also manifest severe defects in insulin-stimulated phosphate transport in skeletal muscle, which may contribute to their observed defects in insulin-stimulated rates of mitochondrial ATP synthesis. Given the potentially important role that intracellular phosphate might have on regulating mitochondrial ATP production, we propose that regulation of intramyocellular P_i_ transport into the muscle cell by insulin may be an important mechanism by which insulin regulates mitochondrial ATP synthesis in skeletal muscle.

**Patient Summary**

**Background**

Type 2 diabetes is one of the fastest growing chronic diseases. We know quite a bit about the risk factors that predispose people to diabetes (such as being overweight), but we do not understand well how the disease starts (usually in adults but increasingly in teenagers and even children).

**Why Was This Study Done?**

Children whose parents are diabetic have a higher risk of developing type 2 diabetes themselves. Some of them have the first signs of the disease (a condition called insulin resistance, which is a strong predictor for subsequent development of diabetes) while they are still young and lean. The group of Gerald Shulman (who is the senior author of this study) has studied such individuals to understand the causes and effects of their insulin resistance. Having previously found some abnormalities in the mitochondria (the parts of cells involved in generating energy), they studied mitochondrial function in more detail here.

**What Did the Researchers Do and Find?**

They compared seven young lean insulin-resistant adults with a family history of diabetes with seven lean young adults without diabetic family members and with normal insulin sensitivity. Specifically, they looked at energy generated in the muscles in response to insulin stimulation. They found that while insulin increases energy production in the muscles of the control individuals by approximately 90%, it had very little effect in the insulin-resistant individuals. They also studied the amount of inorganic phosphate (an essential trace element) in muscle cells of both groups, and how it was affected by insulin. They found that in control individuals, insulin results in an increase of phosphate transport into the muscle cells and that this is also much reduced in insulin-resistant individuals.

**What Does This Mean?**

Intracellular phosphate is a key regulator of energy generation, and this study provides more support for the notion that insulin resistance compromises proper functioning of energy generation in the mitochondria of muscle cells. This and similar studies should eventually result in a better understanding of the early events in diabetes. The hope is that this will then allow the development of therapies that might prevent the disease or treat its symptoms more effectively.

**Where Can I Find More Information Online?**

The following Web sites contain information on diabetes and insulin resistance.

US National Diabetes Clearinghouse (English and Spanish):

http://diabetes.niddk.nih.gov/dm/pubs/insulinresistance/

http://diabetes.niddk.nih.gov/spanish/indexsp.asp

Stanford University Web site on syndrome X, diabetes, and insulin resistance:

http://syndromex.stanford.edu/

Diabetes UK:

http://www.diabetes.org.uk/

American Diabetes Association (English and Spanish):

http://www.diabetes.org/

http://www.diabetes.org/espanol/default.jsp

Information on Dr. Shulman's research:

http://www.hhmi.org/research/investigators/shulman.html

## Abbreviations

ATP: adenosine triphosphate
BMI: body mass index
IMCL: intramyocellular lipid
IR: insulin-resistant
MRS: magnetic resonance spectroscopy
PCr: phosphocreatinine
Pi: inorganic phosphate
